# Factors Associated With End‐of‐Life Care Practice in Nonspecialist Palliative Care Settings: A Capability–Opportunity–Motivation Perspective

**DOI:** 10.1155/jonm/8686334

**Published:** 2026-10-20

**Authors:** Dongyan Zeng, Yinxian Li, Rongzhi Xie, Xiaohua Gong, Shili Chen, Dandan Jiao

**Affiliations:** ^1^ Cancer Center The Fifth Affiliated Hospital Sun Yat-Sen University Zhuhai 519000 China sysu.edu.cn; ^2^ Department of Thoracic Oncology The Fifth Affiliated Hospital Sun Yat-Sen University Zhuhai 519000 China sysu.edu.cn; ^3^ Department of General Practice The Fifth Affiliated Hospital Sun Yat-Sen University Zhuhai 519000 China sysu.edu.cn; ^4^ Department of Abdominopelvic Oncology The Fifth Affiliated Hospital Sun Yat-Sen University Zhuhai 519000 China sysu.edu.cn; ^5^ Department of Nursing The First Affiliated Hospital, and College of Clinical Medicine Henan University of Science and Technology Luoyang 471003 China haust.edu.cn

**Keywords:** COM-B model, competence, end-of-life care, nonspecialist palliative care, nursing practice, outcome expectations

## Abstract

**Background:**

End‐of‐life care is often delivered in nonspecialist palliative care settings. However, challenges in end‐of‐life care practice persist in such settings, and associated factors remain underexplored.

**Aims:**

To examine how capability‐, opportunity‐, and motivation‐related factors are associated with end‐of‐life care practice among nurses and physicians working in nonspecialist palliative care settings.

**Design:**

A cross‐sectional survey.

**Methods:**

Nurses and physicians employed at a tertiary hospital in China and providing direct patient care were recruited. Measures assessed capability‐related factors (end‐of‐life care competence and self‐rated physical health), opportunity‐related factors (social and organizational environments), and motivation‐related factors (outcome expectations and compassion satisfaction), with end‐of‐life care practice as the outcome. Data were analyzed using structural equation modeling.

**Results:**

A total of 342 participants were included in the analysis, of whom 82.7% were nurses. End‐of‐life care competence showed both direct (*β* = 0.413, *p* < 0.001) and indirect associations with end‐of‐life care practice through outcome expectations (*β* = 0.059, 95% CI [0.010, 0.138]). The social environment was indirectly associated with practice through outcome expectations (*β* = 0.082, 95% CI [0.024, 0.189]). Poor or fair self‐rated physical health showed a small negative association with practice through outcome expectations (*β* = −0.022, 95% CI [–0.056, −0.001]). Organizational environment and compassion satisfaction were not significantly associated with practice in the structural model. Overall, the model explained 32% of the variance in end‐of‐life care practice.

**Conclusion:**

End‐of‐life care competence was associated with end‐of‐life care practice, while outcome expectations were involved in the indirect associations of both competence and the social environment with practice. These findings highlight the potential relevance of competence and outcome expectations to end‐of‐life care practice in nonspecialist palliative care settings.

**Implications for Nursing Management:**

Nursing managers may consider prioritizing competency‐based education and continuing professional development, while considering the social environment and healthcare professionals’ outcome expectations related to end‐of‐life care practice.

## 1. Introduction

End‐of‐life care, as a form of palliative care, focuses on relieving suffering, maintaining comfort, and preserving dignity through physical, psychological, social, and spiritual support for patients approaching the end of life and their families [1]. With global population aging and the increasing burden of cancer and other noncommunicable diseases, demand for palliative care is rising rapidly worldwide [2]. Palliative care has been shown to benefit patients and their families by alleviating symptoms, improving quality of life, reducing aggressive end‐of‐life treatments, and lowering healthcare costs [3]. However, despite the growing need and well‐documented benefits, access to and quality of palliative care remain limited, particularly in low‐ and middle‐income countries [2, 4].

In many countries, including China, end‐of‐life care is predominantly delivered in nonspecialist palliative care settings, where specialist hospice units and services remain limited [1]. International palliative care policies increasingly emphasize that palliative care should be embedded into everyday healthcare practice [3]. In this context, nurses and physicians working in nonspecialist palliative care settings play a pivotal role in the provision of end‐of‐life care. Despite this central role, end‐of‐life care practice in nonspecialist palliative care settings remains variable and faces ongoing challenges [5].

Although providing appropriate end‐of‐life care is part of healthcare professionals’ professional responsibilities, its implementation in routine clinical practice may still vary in relation to professionals’ competence, available support, and other behavior‐related factors. Previous research across diverse clinical settings has shown that healthcare professionals’ professional competence (e.g., knowledge and skills), organizational and policy support (e.g., staffing and institutional resources), and broader social contexts (e.g., public perceptions of end‐of‐life care) are associated with end‐of‐life care practice. In addition, emerging evidence suggests that motivational factors, such as expectations regarding the outcomes of end‐of‐life care practice and emotional experiences associated with providing such care, may also be relevant to practice [6–8].

Nevertheless, important gaps remain in the existing literature. Much of the existing evidence has relied on qualitative research to explore barriers and facilitators [9, 10]. This has limited the ability to quantify the relative associations of different factors associated with end‐of‐life care practice and to clarify how these factors are linked to practice, which is important for informing targeted intervention strategies. Moreover, although end‐of‐life care is predominantly delivered in nonspecialist palliative care settings in many health systems, empirical evidence examining end‐of‐life care practice and its associated factors within these routine clinical contexts remains limited.

To address these gaps, the Capability–Opportunity–Motivation–Behavior (COM‐B) model provides a useful framework for examining factors associated with end‐of‐life care practice in nonspecialist palliative care settings and the potential pathways linking these factors. The COM‐B model is well suited to this context as it integrates capability, opportunity, and motivation, aligning closely with factors previously identified as relevant to end‐of‐life care practice. The COM‐B model posits that behavior results from the interaction of three components: Capability, Opportunity, and Motivation [11]. Capability refers to individuals’ psychological and physical capacity to perform a behavior; opportunity reflects external factors that facilitate or hinder behavior; and motivation encompasses the cognitive and emotional processes that influence behavioral engagement.

Guided by the COM‐B model, contextually relevant constructs were selected and mapped onto capability‐, opportunity‐, and motivation‐related domains. Capability‐related constructs included end‐of‐life care competence and self‐rated physical health. Opportunity‐related constructs included social and organizational environments, encompassing government support, public acceptance, organizational support, and resources relevant to end‐of‐life care practice. Motivation‐related constructs included outcome expectations and compassion satisfaction, reflecting expectations regarding the outcomes of end‐of‐life care practice [7] and positive professional satisfaction associated with helping others [6], respectively.

On this basis, this study aimed to examine the associations of capability‐, opportunity‐, and motivation‐related factors with end‐of‐life care practice in nonspecialist palliative care settings and to explore the potential pathways linking these factors with practice. We hypothesized that capability‐, opportunity‐, and motivation‐related factors would be associated with end‐of‐life care practice. Specifically, higher end‐of‐life care competence and better self‐rated physical health, more supportive social and organizational environments, and more positive outcome expectations and greater compassion satisfaction were expected to be associated with higher levels of end‐of‐life care practice. Consistent with the COM‐B framework, we further hypothesized indirect associations of end‐of‐life care competence, self‐rated physical health, social environment, and organizational environment with end‐of‐life care practice through outcome expectations and compassion satisfaction. The hypothesized model is shown in Figure 1.

**FIGURE 1:**
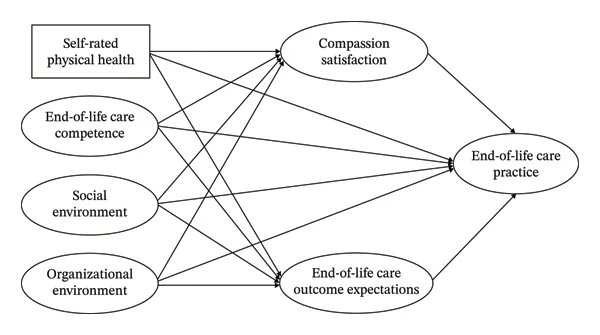
Hypothesized model.

## 2. Materials and Methods

### 2.1. Design

A cross‐sectional survey was employed.

### 2.2. Study Setting and Participants

This survey was conducted among nurses and physicians employed in a 2300‐bed tertiary hospital in Guangdong Province, China. Nurses and physicians were recruited by convenience sampling from general inpatient wards with frequent exposure to patients at end‐of‐life. Eligibility criteria included providing direct patient care; having cared for at least one end‐of‐life patient—defined as a patient with an advanced, life‐limiting illness and an anticipated survival of less than 6 months, based on physicians’ estimation—within the past month; and at least 3 months of clinical experience. Exclusion criteria were trainees without independent practice roles and those on leave during data collection.

### 2.3. Variables and Measurements

All scales used in this study have been used in Chinese healthcare settings and have demonstrated acceptable psychometric properties. Several instruments retained the term “hospice care” in their original titles; however, their content assesses end‐of‐life care practice and related factors relevant to routine clinical settings in which end‐of‐life care is provided.

#### 2.3.1. Sociodemographic and Work‐Related Characteristics

Variables included age, gender, education, marital status, religious belief, profession, years of clinical practice, and clinical department.

#### 2.3.2. Behavior

End‐of‐life care practice was measured with the 14‐item Hospice Care Practice Scale (HCPS) [12], which assesses the frequency of end‐of‐life care behaviors, including communication and care planning, pain and symptom management, psychosocial and spiritual support, care coordination, and family support. Items were rated on a five‐point Likert scale (1 = *never* to 5 = *always*), with higher scores indicating more frequent practice. End‐of‐life care practice was specified as a latent construct in the structural equation model.

#### 2.3.3. Capability‐Related Factors

End‐of‐life care competence was measured with the 21‐item End‐of‐life Professional Caregiver Survey (EPCS) [7], comprising three dimensions: patient‐ and family‐centered communication (PFCC, 5 items), cultural, ethical, and national values (CENV, 11 items), and effective care delivery (ECD, 5 items). Items were rated on a five‐point Likert scale (1 = *strongly disagree* to 5 = *strongly agree*), with higher scores indicating greater competence. In the structural equation model, competence was specified as a second‐order latent construct, represented by its three dimensions.

Self‐rated physical health was assessed using a single‐item measure in which participants rated their overall physical health as good, fair, or poor. This variable was specified as an observed variable in the structural equation model.

#### 2.3.4. Opportunity‐Related Factors

Social and organizational environments were measured with the 13‐item Hospice Care Environment Scale (HCES) [7]. The social environment subscale (4 items) assesses government support and public acceptance, while the organizational environment subscale (9 items) captures staffing, facilities, institutional policies, and multidisciplinary collaboration. Items were rated on a five‐point Likert scale (1 = *strongly disagree* to 5 = *strongly agree*), with higher scores indicating more supportive environments. In structural equation modeling (SEM), both subscales were modeled as separate latent constructs.

#### 2.3.5. Motivation‐Related Factors

End‐of‐life care outcome expectations were measured with the 7‐item Hospice Care Outcome Expectations Scale (HCOES) [7], which assesses expectations regarding personal fulfillment, professional development, workplace relationships, social recognition, and financial outcomes associated with providing end‐of‐life care. Items were rated on a five‐point Likert scale (1 = *strongly disagree* to 5 = *strongly agree*), with higher scores indicating more positive expectations. In the structural equation model, outcome expectations were specified as a latent construct.

Compassion satisfaction was measured with the 10‐item compassion satisfaction subscale of the Professional Quality of Life Scale (ProQOL) [13]. Items are rated on a five‐point Likert scale (1 = *never* to 5 = *very often*), with higher scores reflecting greater satisfaction. Compassion satisfaction was specified as a latent construct in the structural equation model.

### 2.4. Data Collection

Data were collected between July 2024 and August 2025 through both paper‐based questionnaires administered on site and an online survey platform (Wenjuanxing; https://www.wjx.cn/). Questionnaires were self‐administered, required approximately 10–15 min to complete, and were returned anonymously.

A total of 342 valid questionnaires were analyzed, exceeding the commonly recommended minimum sample size of 200 for SEM [14]. Prior simulation studies indicate that, with moderate factor loadings (0.60–0.70) and structural path coefficients (*β* ≈ 0.30–0.40), a sample size of approximately 200 can achieve adequate statistical power with acceptable parameter bias [15]. In the present study, standardized factor loadings ranged from 0.62 to 0.98, and structural path coefficients reached comparable magnitudes (up to *β* = 0.41), supporting the adequacy of the sample size (*N* = 342) for the analyses.

### 2.5. Data Analysis

Descriptive statistics, correlations, internal consistency (Cronbach’s *α*), and normality tests were examined using SPSS 26.0. All main variables showed acceptable normality (|skewness| and |kurtosis| < 1). Common method bias was examined with Harman’s single‐factor test [16]. Missing data were minimal (< 1% for compassion satisfaction and self‐rated physical health) and were imputed using mean and mode substitution, respectively; demographic variables with missing values were handled using listwise deletion in the covariate‐adjusted models, with the highest missingness being 6.1%.

Item parceling was used to reduce the number of indicators and obtain a more parsimonious measurement model [17]. Parceling was applied to the 11‐item CENV dimension of the EPCS, the 10‐item ProQOL compassion satisfaction subscale, and the HCPS. The established three‐dimensional structure of the EPCS was retained, with no items combined across dimensions. The HCPS has been consistently scored and used as an overall measure of end‐of‐life care practice in previous studies [7, 18]; accordingly, it was represented as an overall practice construct. Item‐to‐construct balanced parcels were formed by distributing items with relatively higher and lower factor loadings across parcels [17]. An item‐level sensitivity analysis was conducted to assess whether the structural findings were sensitive to parceling.

We first assessed the measurement model, followed by evaluation of the structural model. Reliability was assessed using Cronbach’s *α* and composite reliability (CR). Convergent validity was evaluated based on significant standardized factor loadings and average variance extracted (AVE). Discriminant validity was assessed using the Fornell–Larcker criterion [19]. Potential multicollinearity among variables in the structural equation for end‐of‐life care practice was assessed using variance inflation factors (VIFs).

The structural model was examined using SEM in Mplus 8.3 version with maximum likelihood estimation. Indirect effects were tested with bias‐corrected bootstrapping (5000 resamples). Model fit was evaluated using the chi‐square test statistic (*χ* ^2^), the comparative fit index (CFI), the Tucker–Lewis index (TLI), the root mean square error of approximation (RMSEA), and the standardized root mean square residual (SRMR), with CFI/TLI ≥ 0.90, RMSEA ≤ 0.08, and SRMR ≤ 0.08 considered acceptable [20]. Models were first estimated without covariates and then re‐estimated adjusting for gender, education, profession, and years of clinical practice, which have been reported to be associated with end‐of‐life care practice [1, 21]. Age was not included due to collinearity with years of clinical practice, and religious belief was not included because of its low prevalence in the sample.

### 2.6. Ethical Considerations

The study was approved by the Ethics Committee of The Fifth Affiliated Hospital, Sun Yat‐sen University (Approval No. K185‐1, 8 July 2024). Participation was voluntary, with informed consent obtained either in writing on site or electronically via the online platform.

## 3. Results

### 3.1. Sample Characteristics

Of 351 questionnaires distributed, 342 valid responses were analyzed. Nine questionnaires were excluded due to substantial missing data across key study variables and implausible response patterns. The mean age of participants was 31.68 years (SD = 6.96). The sample was predominantly composed of nurses (82.7%), with females accounting for 89.2% of all participants. Most participants held a bachelor’s degree (78.7%) and had at least 5 years of clinical practice (60.8%). Participants were recruited from multiple clinical departments within the same tertiary hospital, including oncology wards (29.2%), other medical wards (43.3%), and other inpatient units. Detailed sociodemographic and work‐related characteristics are presented in Table 1.

**TABLE 1:** Sociodemographic and work‐related characteristics of participants (*N* = 342).

Variable	*n* (%)	Variable	*n* (%)
Age (years)		Profession	
< 30	142 (41.5)	Nurse	283 (82.7)
30–39	136 (39.8)	Physician	49 (14.3)
≥ 40	57 (16.7)	Missing	10 (2.9)
Missing	7 (2.0)	Years of clinical practice	
Gender		< 5	113 (33.0)
Female	305 (89.2)	5–10	96 (28.1)
Male	35 (10.2)	> 10	112 (32.7)
Missing	2 (0.6)	Missing	21 (6.1)
Education		Clinical department	
Postgraduate or higher	42 (12.3)	Oncology wards	100 (29.2)
Bachelor	269 (78.7)	Other medical wards	148 (43.3)
Associate or below	27 (7.9)	Surgical wards	19 (5.6)
Missing	4 (1.2)	Critical care units	56 (16.4)
Marital status		Other	12 (3.5)
Married	154 (45.0)	Missing	7 (2.0)
Single	180 (52.6)		
Divorced/widowed	6 (1.8)		
Missing	2 (0.6)		
Religious belief			
Yes	16 (4.7)		
None	325 (95.0)		
Missing	1 (0.3)		

*Note:* Age: mean = 31.68 years (SD = 6.96; range = 21–60).

### 3.2. Descriptions and Correlations of Study Variables

Descriptive statistics for key variables are presented in Table 2. The mean score of end‐of‐life care practice was 52.15 (*SD* = 9.81). At the item level (Supporting Table S1), pain assessment (*M* = 4.31, *SD* = 0.82) and pain relief (*M* = 4.15, *SD* = 0.87) were most frequent, whereas discussing death‐related issues (*M* = 3.27, *SD* = 1.16) and discussing hospice services or referral options (*M* = 2.73, *SD* = 1.22) were least frequent. Regarding self‐rated physical health, 49.7% rated as good, 47.1% as fair, and 2.3% as poor.

**TABLE 2:** Descriptive statistics, construct correlations, and square root of AVE (*N* = 342).

Variable	M	SD	Potential range	1	2	3	4	5	6
1. End‐of‐life care practice	52.15	9.81	14–70	**0.910**					
2. End‐of‐life care competence	65.93	18.06	21–105	0.506^∗∗^	**0.932**				
3. Social environment	14.11	3.34	4–20	0.359^∗∗^	0.608^∗∗^	**0.873**			
4. Organizational environment	31.58	7.60	9–45	0.365^∗∗^	0.638^∗∗^	0.740^∗∗^	**0.846**		
5. End‐of‐life care outcome expectations	26.58	5.42	7–35	0.070	0.016	0.074	0.035	**0.822**	
6. Compassion satisfaction	34.25	7.75	10–50	0.335^∗∗^	0.523^∗∗^	0.514^∗∗^	0.443^∗∗^	0.093	**0.889**
7. Self‐rated physical health	—	—	—	−0.085	−0.082	−0.109^∗^	−0.053	−0.057	−0.240^∗∗^

*Note:* Diagonal elements (in bold) are the square roots of AVE. Off‐diagonal elements represent Pearson correlation coefficients, except for self‐rated physical health, for which Spearman correlations were used due to its ordinal scale (1 = good, 2 = fair, 3 = poor).

Abbreviation: AVE = average variance extracted.

^∗^ *p* < 0.05.

^∗∗^ *p* < 0.01.

Correlation analysis (Table 2) showed that end‐of‐life care practice was positively associated with end‐of‐life care competence, social and organizational environments, and compassion satisfaction (*r* = 0.335–0.506, all *p* < 0.01). End‐of‐life care outcome expectations and self‐rated physical health were not significantly associated with end‐of‐life care practice.

### 3.3. Reliability and Validity of Study Variables

Harman’s single‐factor test indicated that the first factor accounted for 39.7% of the variance, suggesting that common method bias was not substantial. All constructs showed strong reliability, with Cronbach’s *α* and CR ranging from 0.894 to 0.958 (Supporting Table S2). Convergent validity was supported, with AVE values exceeding 0.50 (0.632–0.882) and all standardized factor loadings being significant (0.624–0.984, *p* < 0.001). Discriminant validity was confirmed, as the square roots of AVE were greater than interconstruct correlations (Table 2). The second‐order construct of end‐of‐life care competence was well represented by its three dimensions, with loadings ranging from 0.878 to 0.984 (Supporting Table S3). VIFs ranged from 1.016 to 2.584, indicating no problematic multicollinearity among variables entered into the structural equation for end‐of‐life care practice.

### 3.4. Hypothesized Model Testing

The hypothesized model showed an acceptable overall fit to the data although the TLI value was marginally below the prespecified threshold of 0.90 (*χ* ^2^ (721) = 1745.31, CFI = 0.906, TLI = 0.898, SRMR = 0.056, RMSEA = 0.064). The model (Figure 2) explained 32% of the variance in end‐of‐life care practice, 40% in compassion satisfaction, and 31% in outcome expectations. Standardized coefficients are shown in Supporting Tables S4‐S5. Results remained consistent after adjusting for demographic covariates (Supporting Tables S6‐S7), except that the small indirect effect of self‐rated physical health through outcome expectations was no longer significant. Therefore, unadjusted estimates are presented in the main text.

**FIGURE 2:**
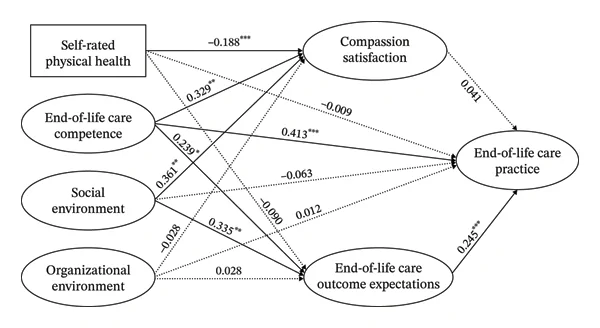
Final model with standardized coefficients. *Note.* Standardized coefficients are presented; dashed lines indicate nonsignificant paths (*p* > 0.05). Self‐rated physical health was coded 1 = fair/poor and 0 = good; negative paths indicate lower outcome expectations, lower compassion satisfaction, and reduced end‐of‐life care practice among those with fair/poor health. ^∗^ *p* < 0.05, ^∗∗^ *p* < 0.01, and ^∗∗∗^ *p* < 0.001.

Among capability‐related factors, end‐of‐life care competence was significantly associated with compassion satisfaction (*β* = 0.329, *p* = 0.001), outcome expectations (*β* = 0.239, *p* = 0.035), and practice (*β* = 0.413, *p* < 0.001). Self‐rated physical health was dichotomized (fair/poor vs. good), and participants with fair/poor health reported lower compassion satisfaction (*β* = −0.188, *p* < 0.001) and marginally lower outcome expectations (*β* = −0.090, *p* = 0.056). Among opportunity‐related factors, social environment was significantly associated with compassion satisfaction (*β* = 0.361, *p* = 0.001) and outcome expectations (*β* = 0.335, *p* = 0.008), whereas organizational environment showed no significant direct associations. Among motivation‐related factors, outcome expectations were significantly associated with end‐of‐life care practice in the structural model (*β* = 0.245, *p* < 0.001), whereas compassion satisfaction was not significantly associated with practice (*β* = 0.041, *p* = 0.631).

Bootstrap analyses indicated significant indirect associations of competence (*β* = 0.059, 95% CI [0.010, 0.138]) and social environment (*β* = 0.082, 95% CI [0.024, 0.189]) with practice through outcome expectations. Fair/poor self‐rated physical health (vs. good) showed a small negative indirect association with practice through outcome expectations (*β* = −0.022, 95% CI [–0.056, −0.001]). No significant indirect associations through compassion satisfaction were observed, and organizational environment showed no significant indirect association with practice.

Sensitivity analysis using an item‐level SEM of the same hypothesized model yielded a pattern of structural associations consistent with that of the parcel‐based model. The item‐level model met the prespecified criteria for RMSEA and SRMR (RMSEA = 0.068, SRMR = 0.069), whereas CFI and TLI were below the prespecified threshold of 0.90 (CFI = 0.838, TLI = 0.832). The pattern of significant and nonsignificant direct associations was consistent with that of the parcel‐based model, and the same indirect associations through outcome expectations remained significant (Supporting Tables S8‐S9).

## 4. Discussion

This study applied the COM‐B model to examine factors associated with end‐of‐life care practice among nurses and physicians working in nonspecialist palliative care settings. End‐of‐life care competence emerged as the strongest factor associated with practice, with both direct and indirect associations through end‐of‐life care outcome expectations. Outcome expectations were involved in the indirect associations of end‐of‐life care competence and social environment with end‐of‐life care practice. Overall, these findings highlight the importance of strengthening end‐of‐life care competence while considering the roles of the social environment and healthcare professionals’ outcome expectations in supporting end‐of‐life care practice in nonspecialist palliative care settings.

Consistent with a growing body of international evidence indicating that insufficient end‐of‐life care competence constitutes a key barrier to the delivery of end‐of‐life care in clinical practice [22], this study underscores the importance of end‐of‐life care competence in end‐of‐life care practice among nurses and physicians working in nonspecialist palliative care settings. Beyond its direct association with practice, end‐of‐life care competence also showed an indirect association with practice through outcome expectations. From the perspective of Bandura’s social cognitive theory, greater competence may be associated with greater confidence in one’s capability to provide end‐of‐life care, which may in turn contribute to more positive outcome expectations [23]. Outcome expectations, an important motivational construct in social cognitive theory, may, therefore, help explain the observed indirect association between end‐of‐life care competence and practice.

Notably, symptom management behaviors (e.g., pain assessment and relief) were reported most frequently, whereas death‐related communication and hospice referral practices were reported least frequently in this study. This pattern is consistent with both Chinese [7] and international findings [24]. Evidence from nonspecialist palliative care settings further suggests that healthcare professionals generally demonstrate relatively greater knowledge and positive attitudes toward symptom management, yet experience greater difficulty and lower confidence in death‐related communication [1]. Death‐related communication and hospice referral practices place high demands on professional competence, particularly in communication skills, ethical reasoning, and the ability to manage clinical uncertainty. Previous studies have reported limited availability and coverage of palliative care education and training in these nonspecialist palliative care settings [5]. Moreover, even when training is available, it often lacks sufficient depth and clinical relevance, particularly with respect to end‐of‐life communication, as has been observed in the Chinese context [8, 25]. Together, these factors may partly explain why symptom management behaviors were more commonly reported than communication‐ and referral‐related practices in this study.

In this study, outcome expectations were involved in the indirect associations of end‐of‐life care competence and social environment with end‐of‐life care practice. Beyond individual competence, our findings suggest that the social environment may be associated with outcome expectations by conveying whether end‐of‐life care is socially supported and valued. A supportive social environment—as reflected in policy endorsement and public acceptance—may be associated with more positive outcome expectations among healthcare professionals, potentially reflecting greater recognition of the professional meaning and value of end‐of‐life care. The observed indirect association between the social environment and end‐of‐life care practice through outcome expectations may be particularly relevant in contexts where end‐of‐life care is not yet widely accepted in the general public and discussions of death remain culturally sensitive, such as in China. In such contexts, greater societal recognition and acceptance of end‐of‐life care may provide a supportive context for healthcare professionals to recognize the value of end‐of‐life care practice.

Furthermore, although outcome expectations were not significantly correlated with end‐of‐life care practice at the bivariate level, they were significantly associated with practice in the structural model after other capability‐, opportunity‐, and motivation‐related factors were considered. This difference may reflect a suppression effect. VIFs ranged from 1.016 to 2.584, providing no evidence of problematic multicollinearity among variables entered into the structural equation for end‐of‐life care practice. These findings suggest that the association between outcome expectations and practice may be better interpreted in the context of the other factors included in the structural model and with appropriate caution.

Although self‐rated physical health showed a small indirect association with end‐of‐life care practice through outcome expectations, this association was no longer significant after adjustment for demographic covariates and should, therefore, be interpreted cautiously. Organizational environment and compassion satisfaction were not significantly associated with end‐of‐life care practice within the pathways examined in the present model, suggesting that further research is needed to clarify their potential roles and the conditions under which they may be relevant to practice.

### 4.1. Strengths and Limitations of the Work

A key strength of this study lies in its application of the COM‐B framework within a quantitative design to examine capability‐, opportunity‐, and motivation‐related factors associated with end‐of‐life care practice, including their indirect associations. By examining nurses and physicians in a nonspecialist palliative care setting, the findings offer insights for nursing management of end‐of‐life care in routine clinical contexts.

Several limitations should be noted. First, this study was conducted in a single tertiary hospital in China, which may limit the generalizability of the findings to other settings and contexts. Second, the overall model fit should be interpreted with caution. The TLI of the hypothesized model and both the CFI and TLI of the covariate‐adjusted model were slightly below the prespecified threshold of 0.90, indicating that the models did not fully meet all prespecified fit criteria. Third, end‐of‐life care practice and other study variables were assessed using self‐reported measures, which may be subject to social desirability or recall bias. Finally, the cross‐sectional design precludes causal inference and limits conclusions about temporal relationships.

### 4.2. Implications for Further Research

Future research may extend this work in several ways. First, multicenter studies across different healthcare institutions within nonspecialist palliative care settings are needed to examine the generalizability of these findings. Second, future studies should examine additional theoretically relevant constructs and alternative structural pathways to further refine the model of healthcare professionals’ end‐of‐life care practice. Third, multisource assessments, such as patient or family reports and objective indicators, could be incorporated to reduce reliance on self‐reported data. Finally, longitudinal studies are needed to clarify the temporal ordering of the observed associations, while appropriately designed intervention studies could further examine causal relationships.

### 4.3. Implications for Nursing Management

Given that nurses comprised 82.7% of the sample, the findings have particular relevance for nursing management and education. Nursing managers may consider prioritizing competency‐based education and continuing professional development to strengthen nurses’ end‐of‐life care competence, particularly in nonspecialist palliative care settings. Educational and training approaches may focus on clinically challenging aspects of practice, such as end‐of‐life communication, ethical decision‐making, and hospice referral. Such approaches may help nurses develop greater confidence and preparedness for end‐of‐life care and support its integration into routine clinical practice.

Beyond competence development, nursing managers may also consider strategies that support positive outcome expectations related to end‐of‐life care practice. From a broader system perspective, nursing leaders may advocate for supportive health policies and public education initiatives that enhance societal acceptance and recognition of end‐of‐life care, given the observed association between the social environment and healthcare professionals’ outcome expectations.

## 5. Conclusion

This study applied the COM‐B model to examine factors and pathways associated with end‐of‐life care practice among nurses and physicians working in nonspecialist palliative care settings, with nurses comprising the majority of the sample. End‐of‐life care competence emerged as the strongest factor associated with practice, showing both direct and indirect associations through outcome expectations.

Outcome expectations were involved in the indirect associations of end‐of‐life care competence and the social environment with end‐of‐life care practice. Together, these findings highlight the importance of end‐of‐life care competence and the potential relevance of outcome expectations and the social environment to end‐of‐life care practice in nonspecialist clinical settings.

## Funding

This research received no specific grant from any funding agency in the public, commercial, or not‐for‐profit sectors.

## Conflicts of Interest

The authors declare no conflicts of interest.

## Supporting Information

Additional supporting information can be found online in the Supporting Information section.

## Supporting information


**Supporting Information** The supporting information includes nine supporting tables (> Tables S1–S9) providing additional information related to the study measures and analyses.

## Data Availability

The data are available from the corresponding authors upon reasonable request.
