# Supplementary material for: Factors Associated With End‐of‐Life Care Practice in Nonspecialist Palliative Care Settings: A Capability–Opportunity–Motivation Perspective
Source: J Nurs Manag. 2026 Oct 20;2026:8686334. doi: 10.1155/jonm/8686334 (PMC13684061; doi:10.1155/jonm/8686334)
Supplement: Supplementary file 1 [file JONM-2026-8686334-s001.docx]

**Supplementary File**

Table S1

Descriptive Statistics of End-of-life Care Practice Items (N = 342)

| Item | M | SD |
| --- | --- | --- |
| 1. Discuss death-related issues with patients and families | 3.27 | 1.16 |
| 2. Discuss hospice services or referral options with patients and families | 2.73 | 1.22 |
| 3. Emphasize respect for patients’ wishes | 3.62 | 1.11 |
| 4. Relieve patients’ pain and discomfort | 4.15 | 0.87 |
| 5. Conduct pain assessments | 4.31 | 0.82 |
| 6. Avoid or reduce unnecessary medical treatments and expenses | 3.96 | 0.90 |
| 7. Address patients’ physical and psychological needs | 4.08 | 0.83 |
| 8. Explain the expected dying process to patients and families | 3.40 | 1.18 |
| 9. Guide families on providing meaningful support for patients | 3.80 | 0.91 |
| 10. Recognize families’ needs and address their suffering | 3.96 | 0.87 |
| 11. Build supportive relationships with families | 4.05 | 0.84 |
| 12. Coordinate medical, social, psychological, and spiritual resources | 3.57 | 1.03 |
| 13. Support bereaved families at high risk for grief | 3.66 | 0.95 |
| 14. Provide guidance to families regarding post-death arrangements | 3.59 | 1.05 |

*Note. Items were rated on a 5-point Likert scale (1 = Never, 5 = Always), with higher scores indicating more frequent practice.*

Table S2

Construct Reliability and Validity of Study Variables (N = 342)

| Variable | Cronbach’s α | CR | AVE |
| --- | --- | --- | --- |
| End-of-life care practice | 0.923 | 0.935 | 0.828 |
| End-of-life care competence (2nd-order) | – | 0.952 | 0.868 |
| Patient- and family-centered communication (PFCC) | 0.904 | 0.906 | 0.658 |
| Cultural, ethical, and national values (CENV) | 0.958 | 0.957 | 0.882 |
| Effective care delivery (ECD) | 0.894 | 0.894 | 0.632 |
| Social environment | 0.926 | 0.928 | 0.762 |
| Organizational environment | 0.957 | 0.958 | 0.715 |
| End-of-life care outcome expectations | 0.928 | 0.935 | 0.676 |
| Compassion satisfaction | 0.920 | 0.919 | 0.791 |

*Note. CR = composite reliability; AVE = average variance extracted. End-of-life care competence was modeled as a second-order construct, comprising three first-order dimensions: PFCC, CENV, and ECD.*

Table S3

Standardized Factor Loadings for Measurement Items (N = 342)

| Variable | Indicator | β | SE | t/Z | p |
| --- | --- | --- | --- | --- | --- |
| End-of-life care practice | Item parcel 1 | 0.945 | 0.014 | 68.759 | <0.001 |
|  | Item parcel 2 | 0.895 | 0.016 | 56.887 | <0.001 |
|  | Item parcel 3 | 0.889 | 0.015 | 59.628 | <0.001 |
| End-of-life care competence | PFCC | 0.930 | 0.017 | 53.231 | <0.001 |
|  | CENV | 0.984 | 0.012 | 80.384 | <0.001 |
|  | ECD | 0.878 | 0.021 | 40.936 | <0.001 |
| PFCC | Item 1 | 0.813 | 0.025 | 32.305 | <0.001 |
|  | Item 2 | 0.839 | 0.023 | 36.992 | <0.001 |
|  | Item 3 | 0.805 | 0.025 | 31.907 | <0.001 |
|  | Item 4 | 0.775 | 0.028 | 27.280 | <0.001 |
|  | Item 5 | 0.821 | 0.023 | 35.940 | <0.001 |
| CENV | Item parcel 1 | 0.917 | 0.012 | 78.914 | <0.001 |
|  | Item parcel 2 | 0.967 | 0.006 | 158.839 | <0.001 |
|  | Item parcel 3 | 0.932 | 0.013 | 71.553 | <0.001 |
| ECD | Item 1 | 0.624 | 0.044 | 14.040 | <0.001 |
|  | Item 2 | 0.865 | 0.019 | 46.373 | <0.001 |
|  | Item 3 | 0.776 | 0.033 | 23.693 | <0.001 |
|  | Item 4 | 0.887 | 0.018 | 49.047 | <0.001 |
|  | Item 5 | 0.795 | 0.025 | 32.203 | <0.001 |
| Social environment | Item 1 | 0.844 | 0.027 | 31.128 | <0.001 |
|  | Item 2 | 0.899 | 0.015 | 58.670 | <0.001 |
|  | Item 3 | 0.897 | 0.020 | 44.042 | <0.001 |
|  | Item 4 | 0.851 | 0.029 | 29.474 | <0.001 |
| Organizational environment | Item 1 | 0.886 | 0.015 | 57.271 | <0.001 |
|  | Item 2 | 0.885 | 0.020 | 45.243 | <0.001 |
|  | Item 3 | 0.884 | 0.016 | 56.060 | <0.001 |
|  | Item 4 | 0.872 | 0.024 | 36.739 | <0.001 |
|  | Item 5 | 0.803 | 0.035 | 22.712 | <0.001 |
|  | Item 6 | 0.842 | 0.020 | 41.412 | <0.001 |
|  | Item 7 | 0.827 | 0.023 | 36.021 | <0.001 |
|  | Item 8 | 0.835 | 0.019 | 42.931 | <0.001 |
|  | Item 9 | 0.769 | 0.026 | 29.124 | <0.001 |
| End-of-life care outcome expectations | Item 1 | 0.804 | 0.026 | 30.746 | <0.001 |
|  | Item 2 | 0.839 | 0.020 | 42.104 | <0.001 |
|  | Item 3 | 0.856 | 0.021 | 41.139 | <0.001 |
|  | Item 4 | 0.867 | 0.024 | 36.173 | <0.001 |
|  | Item 5 | 0.906 | 0.017 | 52.668 | <0.001 |
|  | Item 6 | 0.820 | 0.036 | 22.667 | <0.001 |
|  | Item 7 | 0.633 | 0.044 | 14.355 | <0.001 |
| Compassion satisfaction | Item parcel 1 | 0.913 | 0.018 | 50.262 | <0.001 |
|  | Item parcel 2 | 0.851 | 0.031 | 27.641 | <0.001 |
|  | Item parcel 3 | 0.903 | 0.016 | 56.809 | <0.001 |

*Note. β = standardized loading; SE = standard error; PFCC = Patient- and family-centered communication; CENV = Cultural, ethical, and national values; ECD = Effective care delivery.*

*End-of-life care competence was modeled as a second-order construct, comprising three first-order dimensions: PFCC, CENV, and ECD. Item parcels were created using factorial balanced method.*

Table S4

Standardized Direct Effects of the Model (N = 342)

| Dependent Variable | Predictor | β | SE | t/Z | p |
| --- | --- | --- | --- | --- | --- |
| Compassion satisfaction | End-of-life care competence | 0.329 | 0.097 | 3.379 | 0.001 |
|  | Social environment | 0.361 | 0.106 | 3.416 | 0.001 |
|  | Organizational environment | －0.028 | 0.101 | －0.276 | 0.783 |
|  | Self-rated physical health (fair/poor) | －0.188 | 0.045 | －4.154 | <0.001 |
| End-of-life care outcome expectations | End-of-life care competence | 0.239 | 0.113 | 2.106 | 0.035 |
|  | Social environment | 0.335 | 0.126 | 2.661 | 0.008 |
|  | Organizational environment | 0.028 | 0.136 | 0.206 | 0.837 |
|  | Self-rated physical health (fair/poor) | －0.090 | 0.047 | －1.913 | 0.056 |
| End-of-life care practice | End-of-life care competence | 0.413 | 0.076 | 5.460 | <0.001 |
|  | End-of-life care outcome expectations | 0.245 | 0.062 | 3.954 | <0.001 |
|  | Compassion satisfaction | 0.041 | 0.086 | 0.481 | 0.631 |
|  | Social environment | －0.063 | 0.111 | －0.570 | 0.569 |
|  | Organizational environment | 0.012 | 0.104 | 0.112 | 0.911 |
|  | Self-rated physical health (fair/poor) | －0.009 | 0.049 | －0.186 | 0.852 |

*Note. β = standardized path coefficient; SE = standard error. Self-rated physical health was dummy coded (0 = good [reference], 1 = fair/poor; “poor” was combined with “fair” due to low prevalence).*

Table S5

Standardized Indirect Effects of the Model (N = 342)

| Indirect Path | β | 95% BC bootstrap CI | Significant |
| --- | --- | --- | --- |
| End-of-life care competence → End-of-life care practice (total) | 0.072 | [0.001, 0.176] | Yes |
| via Compassion satisfaction | 0.014 | [－0.041, 0.079] | No |
| via End-of-life care outcome expectations | 0.059 | [0.010, 0.138] | Yes |
| Social environment → End-of-life care practice (total) | 0.097 | [0.021, 0.220] | Yes |
| via Compassion satisfaction | 0.015 | [－0.042, 0.095] | No |
| via End-of-life care outcome expectations | 0.082 | [0.024, 0.189] | Yes |
| Organizational environment → End-of-life care practice (total) | 0.006 | [－0.072, 0.088] | No |
| via Compassion satisfaction | －0.001 | [－0.036, 0.013] | No |
| via End-of-life care outcome expectations | 0.007 | [－0.059, 0.086] | No |
| Self-rated physical health (fair/poor) → End-of-life care practice (total) | －0.030 | [－0.077, 0.008] | No |
| via Compassion satisfaction | －0.008 | [－0.043, 0.023] | No |
| via End-of-life care outcome expectations | －0.022 | [－0.056, －0.001] | Yes |

*Note. β = standardized path coefficient; 95% BC bootstrap CI = 95% bias-corrected bootstrap confidence interval (5,000 resamples). Self-rated physical health was dummy coded (0 = good [reference], 1 = fair/poor; “poor” was combined with “fair” due to low prevalence). Effects are significant when the CI does not include zero.*

Table S6

Standardized Direct Effects of the Covariate-Adjusted Model (N = 316)

| Dependent Variable | Predictor | β | SE | t/Z | p |
| --- | --- | --- | --- | --- | --- |
| Compassion satisfaction | End-of-life care competence | 0.435 | 0.095 | 4.604 | <0.001 |
|  | Social environment | 0.340 | 0.107 | 3.177 | 0.001 |
|  | Organizational environment | －0.089 | 0.101 | －0.878 | 0.380 |
|  | Self-rated physical health (fair/poor) | －0.204 | 0.045 | －4.493 | <0.001 |
| End-of-life care outcome expectations | End-of-life care competence | 0.298 | 0.111 | 2.687 | 0.007 |
|  | Social environment | 0.299 | 0.127 | 2.359 | 0.018 |
|  | Organizational environment | －0.003 | 0.138 | －0.023 | 0.982 |
|  | Self-rated physical health (fair/poor) | －0.077 | 0.049 | －1.550 | 0.121 |
| End-of-life care practice | End-of-life care competence | 0.430 | 0.082 | 5.223 | <0.001 |
|  | End-of-life care outcome expectations | 0.264 | 0.062 | 4.278 | <0.001 |
|  | Compassion satisfaction | 0.018 | 0.092 | 0.200 | 0.842 |
|  | Social environment | －0.086 | 0.116 | －0.746 | 0.456 |
|  | Organizational environment | 0.046 | 0.103 | 0.450 | 0.653 |
|  | Self-rated physical health (fair/poor) | －0.061 | 0.049 | －1.249 | 0.212 |
|  | Gender (female) | 0.006 | 0.053 | 0.105 | 0.916 |
|  | Education (postgraduate) | 0.136 | 0.068 | 2.007 | 0.045 |
|  | Education (associate or below) | －0.062 | 0.059 | －1.048 | 0.295 |
|  | Profession (nurse) | －0.065 | 0.077 | －0.848 | 0.396 |
|  | Years of clinical practice (5－10) | 0.047 | 0.052 | 0.889 | 0.374 |
|  | Years of clinical practice (> 10) | 0.009 | 0.050 | 0.178 | 0.859 |

*Note. β = standardized path coefficient; SE = standard error. Self-rated physical health was dummy coded (0 = good [reference], 1 = fair/poor; “poor” was combined with “fair” due to low prevalence). Covariates were dummy coded with the following reference groups: gender = male, education = bachelor, profession = physician, years of clinical practice = <5.*

*Model fit: χ² (987) = 2123.96, RMSEA = 0.060, CFI = 0.892, TLI = 0.885, SRMR = 0.068. Analyses were based on 316 participants due to listwise deletion of missing covariate data.*

Table S7

Standardized Indirect Effects of the Covariate-Adjusted Model (N = 316)

| Indirect Path | β | 95% BC bootstrap CI | Significant |
| --- | --- | --- | --- |
| End-of-life care competence → End-of-life care practice (total) | 0.087 | [0.007, 0.203] | Yes |
| via Compassion satisfaction | 0.008 | [－0.076, 0.089] | No |
| via End-of-life care outcome expectations | 0.079 | [0.024, 0.170] | Yes |
| Social environment → End-of-life care practice (total) | 0.085 | [0.003, 0.215] | Yes |
| via Compassion satisfaction | 0.006 | [－0.059, 0.082] | No |
| via End-of-life care outcome expectations | 0.079 | [0.017, 0.183] | Yes |
| Organizational environment → End-of-life care practice (total) | －0.002 | [－0.088, 0.080] | No |
| via Compassion satisfaction | －0.002 | [－0.043, 0.017] | No |
| via End-of-life care outcome expectations | －0.001 | [－0.078, 0.077] | No |
| Self-rated physical health (fair/poor) → End-of-life care practice (total) | －0.024 | [－0.076, 0.021] | No |
| via Compassion satisfaction | －0.004 | [－0.044, 0.035] | No |
| via End-of-life care outcome expectations | －0.020 | [－0.056, 0.004] | No |

*Note. β = standardized path coefficient; 95% BC bootstrap CI = 95% bias-corrected bootstrap confidence interval (5,000 resamples). Self-rated physical health was dummy coded (0 = good [reference], 1 = fair/poor; “poor” was combined with “fair” due to low prevalence). Effects are significant if the CI does not include zero. Analyses were based on 316 participants due to listwise deletion of missing covariate data.*

Table S8

Comparison of Standardized Direct Effects Between the Parcel-Based and Item-Level SEMs (N = 342)

| Dependent variable | Predictor | Parcel-based SEM  β (p) | Item-level SEM  β (p) |
| --- | --- | --- | --- |
| Compassion satisfaction | End-of-life care competence | 0.329 (0.001) | 0.351 (<0.001) |
|  | Social environment | 0.361 (0.001) | 0.336 (0.002) |
|  | Organizational environment | −0.028 (0.783) | −0.028 (0.790) |
|  | Self-rated physical health | −0.188 (<0.001) | −0.186 (<0.001) |
| End-of-life care outcome expectations | End-of-life care competence | 0.239 (0.035) | 0.248 (0.026) |
|  | Social environment | 0.335 (0.008) | 0.324 (0.010) |
|  | Organizational environment | 0.028 (0.837) | 0.032 (0.813) |
|  | Self-rated physical health | −0.090 (0.056) | −0.089 (0.059) |
| End-of-life care practice | End-of-life care competence | 0.413 (<0.001) | 0.380 (<0.001) |
|  | End-of-life care outcome expectations | 0.245 (<0.001) | 0.253 (<0.001) |
|  | Compassion satisfaction | 0.041 (0.631) | 0.043 (0.617) |
|  | Social environment | −0.063 (0.569) | −0.032 (0.788) |
|  | Organizational environment | 0.012 (0.911) | 0.023 (0.840) |
|  | Self-rated physical health | −0.009 (0.852) | −0.017 (0.735) |

*Note. SEM = structural equation modeling;* *β = standardized path coefficient. Self-rated physical health was dummy coded (0 = good [reference], 1 = fair/poor; “poor” was combined with “fair” due to low prevalence).*

*Model fit: χ² (2060) = 5288.19, RMSEA = 0.068, CFI = 0.838, TLI = 0.832, SRMR = 0.069.*

Table S9

Comparison of Standardized Indirect Effects Between the Parcel-Based and Item-Level SEMs (N = 342)

| Indirect pathway | Parcel-based SEM  β (95% BC bootstrap CI) | Item-level SEM  β (95% BC bootstrap CI) |
| --- | --- | --- |
| End-of-life care competence → Compassion satisfaction → End-of-life care practice | 0.014 [−0.041, 0.079] | 0.015 [−0.044, 0.085] |
| End-of-life care competence → End-of-life care outcome expectations → End-of-life care practice | 0.059 [0.010, 0.138] | 0.063 [0.011, 0.143] |
| Social environment → Compassion satisfaction → End-of-life care practice | 0.015 [−0.042, 0.095] | 0.015 [−0.038, 0.089] |
| Social environment → End-of-life care outcome expectations → End-of-life care practice | 0.082 [0.024, 0.189] | 0.082 [0.024, 0.171] |
| Organizational environment → Compassion satisfaction → End-of-life care practice | −0.001 [−0.036, 0.013] | −0.001 [−0.036, 0.012] |
| Organizational environment → End-of-life care outcome expectations → End-of-life care practice | 0.007 [−0.059, 0.086] | 0.008 [−0.059, 0.086] |
| Self-rated physical health → Compassion satisfaction → End-of-life care practice | −0.008 [−0.043, 0.023] | −0.008 [−0.043, 0.022] |
| Self-rated physical health → End-of-life care outcome expectations → End-of-life care practice | −0.022 [−0.056, −0.001] | −0.022 [−0.058, −0.001] |

*Note. SEM = structural equation modeling;* *β = standardized path coefficient; 95% BC bootstrap CI = 95% bias-corrected bootstrap confidence interval (5,000 resamples). Self-rated physical health was dummy coded (0 = good [reference], 1 = fair/poor; “poor” was combined with “fair” due to low prevalence).*
